# Hypometria of saccadic eye movements to targets in rapid circular motion

**DOI:** 10.1167/jov.24.1.2

**Published:** 2024-01-03

**Authors:** Reza Azadi, Alex O. Holcombe, Jay A. Edelman

**Affiliations:** 1 Doctoral Program in Biology, CUNY Graduate Center, New York, NY, USA; 2 The University of Sydney, School of Psychology, Sydney, Australia; 3 Department of Biology, The City College of New York, New York, NY, USA; 4 Doctoral Program in Psychology, CUNY Graduate Center, New York, NY, USA

**Keywords:** saccade, voluntary, timing, tracking

## Abstract

Saccades to objects moving on a straight trajectory take the velocity of the object into account. However, it is not known whether saccades can compensate for curved trajectories, nor is it known how they are affected by high target speeds. In Experiment 1, participants made a saccade in a delayed saccade task to a target moving in a circular trajectory. Surprisingly, saccades to high-speed moving targets were severely hypometric, with gains of only ∼55% for trajectories of the largest angular speed (2 revolutions per second) and eccentricity (12°). They also had unusually low peak velocities. In Experiment 2, the target jumped along a circular path around a central fixation point. Hypometria was still severe, except for very large jumps. Experiment 3 was like Experiment 1, except that a landmark was positioned on the trajectory of the target, and participants were instructed to make a saccade to the landmark or to its memorized location. This ameliorated hypometria considerably. Given the delayed nature of the tasks of Experiments 1 and 2, participants had considerable time to program a voluntary saccade to a location on the trajectory, if not to the rapidly moving target itself. Nevertheless, the abnormal saccade properties indicate that motor programming was compromised. These results indicate that motor output can be inextricably bound to sensory input to its detriment, even during a highly voluntary motor act; that apparent motion can produce this behavior; and that such abnormal saccades can be “rescued” by the presence of a stable visual goal.

## Introduction

The world seldom stands still; thus, interacting with the world often requires keeping track of and orienting to moving things. Orienting to moving visual objects requires not only the ability to perceive them but also, because of nervous system delays and physical limits to action, the use of target velocity for programming movements. The relative simplicity of the eye movement system has made it a model system for understanding how motion information can be incorporated into movement commands. Several studies have shown that the velocity of moving targets can be taken into account during saccade programming by both humans and monkeys. However, such studies all used targets moving along linear trajectories at modest speeds (Etchells, Benton, Ludwig, & Gilchrist, 2010; Fleuriet, Hugues, Perrinet, & Goffart, 2011; Gellman & Carl, 1991; Guan, Eggert, Bayer, & Büttner, 2005; Keller, Gandhi, & Weir, 1996; Keller & Johnsen, 1990; Ron, Vieville, & Droulez, 1989).

Experiment 1 of the present study was designed to assess whether the errors in saccadic eye movement programming were dependent on the speed of targets moving in circular trajectories in a manner similar to that of perceptual judgments. For perceptual judgments, one investigation of the effect of target speed on position judgments of objects moving about fixation was conducted by Linares, Holcombe, and White (2009). At a random time during each trial in this study, a cue such as a sound or light was presented, and the observer reported the perceived position of the target at the time of the cue. Participants perceive a moving object to be further along its trajectory than it actually was at the time of the flash (Nijhawan, 1994). The magnitude of this flash-lag effect increases with speed (Khurana & Nijhawan, 1995; Krekelberg & Lappe, 1999; Linares et al., 2009; Nijhawan, 1994), and each of the studies mentioned here found the increase in spatial magnitude of the effect to be approximately linear, which made it constant in temporal units, suggesting that the effect is temporal. That is, perception makes a temporal error of mistakenly judging the position of the object based on where it is approximately 80 ms after the flash. A popular theory is that this reflects a neural extrapolation process that compensates for sensory neural latency (Nijhawan, 1994), to allow successful interaction with moving objects (Nijhawan, 2008), which predicts that saccades to moving objects should land on their targets. Alternatively, however, some researchers favor other explanations, such as that the flash triggers a time-consuming position sample (Schlag & Schlag-Rey, 2002).

The linear increase in the flash-lag effect with speed was observed up to speeds higher than seem to have been explored with saccades to moving objects (Etchells et al., 2010; Fleuriet et al., 2011; Gellman & Carl, 1991; Guan et al., 2005; Keller & Johnsen, 1990; Keller et al., 1996; Ron et al., 1989), and we wondered whether saccade landing points would, like perception, be ahead by a temporally constant amount over this range of speeds. However, a study of the flash-lag effect that used ultra-fast speeds upwards of 15°/s documented a leveling off of the spatial magnitude (Wojtach, Sung, Truong, & Purves, 2008). Wojtach et al. (2008) suggested that this reflected the probability distribution of image speeds on the retina—in other words, the low prior for very high speeds means that the visual system may estimate speed to be much lower than it actually is.

Another characteristic of the flash-lag effect is large trial-to-trial variability. Linares et al. (2009) found that the variability of the reported positions increased with the speed of the object, such that 1 *SD* of the distribution of position reports corresponded to the distance that the object traveled in about 70 ms. In other words, the variability of the flash-lag effect appears to be constant in temporal units, becoming increasing linearly with speed, just as the magnitude of the bias does (for low speeds, as reviewed in the previous paragraph). Murakami (2001) found similar results with a related task. We sought to investigate this for saccades, as we are not aware of any investigations of whether landing points of saccades to moving objects similarly have variability that increases linearly with speed.

In the localization judgment tasks of Linares et al. (2009), participants had to report the location of the object at the time of a cue, and performance could be only as good as the temporal precision in sampling the object position in response to the cue. When the object was moving quickly, temporal noise involved in binding the cue to the moving object may have been the largest contributor to the variance in reported object position. In contrast, in a saccade task characterizing such localization performance, the participant would simply make a saccade to the moving object, taking into account its motion, with no need to reference the timing of the saccade cue. Performance in a saccade task may thus have different characteristics than explicit perceptual localization. It is also arguably a more ecological task that has the potential to reveal more about the neural processing of the position of a moving object.

The quality of saccadic targeting can be quantified by both bias and variability. Bias, quantified as the mean error, could occur as a result of under- or over-extrapolation or could be a simple hypometria or hypermetria effect. Variability around the mean includes both spatial imprecision and temporal imprecision. *Spatial imprecision* reflects the variability inherent to targeting a static object, and temporal imprecision might result from uncertainty in taking target velocity into account during saccade generation.

In the present study, saccades were made after an instructed delay to stimuli moving in circular trajectories of varying speeds and eccentricities. Saccade amplitude and direction error were measured, as was the velocity and acceleration of saccades. To our surprise, saccades to moving targets were severely hypometric, falling far short of the target path, with amplitude approximately 55% of the target eccentricity for the highest target angular speeds. Such hypometria both undermined our initial experiment aim and motivated two follow-up experiments investigating possible explanations of this unexpected finding.

In Experiment 2, we examined whether severe saccade hypometria to rapidly moving stimuli would persist if the moving stimulus were presented at discrete, spatially separated locations rather than in a continuous motion stream. We hypothesized that presenting the moving stimulus at a succession of discrete locations (in apparent motion rather than continuous motion) would foster more robust representations of individual locations for targeting, enhancing the subject's ability to program a saccade with the correct amplitude. But, the results for stimuli in apparent motion were similar to those in continuous motion. Evidently, rapid change in position of the target, whether continuous or discrete, is sufficient to cause saccade hypometria.

 We then asked whether hypometria results from targeting the rapidly moving stimulus itself or is instead a consequence of having a rapidly moving stimulus in the neighborhood of the saccade goal. To investigate this question, in Experiment 3 we modified the task of Experiment 1 by specifying a stable “goal” location along the circular trajectory. The subjects were told to intercept the moving target at the goal by making a saccade to the goal at the appropriate time. This improved saccade performance, suggesting that the saccade hypometria found in the first two experiments was mainly a result of targeting the rapidly moving stimulus and cannot be attributed entirely to the mere presence of nearby motion.

## Methods

### Subjects, eye movement recording, and visual stimulus display

Five subjects (ages 20–48 years; three naïve and two authors) participated in all three experiments described below. Experiments were conducted under a protocol approved by The City College of New York Institutional Review Board. Movements of the right eye were recorded using an EyeLink II video-based eye tracker (SR Research, Kanata, ON, Canada), sampling at 500 Hz. Stimuli were presented on a CRT monitor (P1220; Compaq, Palo Alto, CA) with a refresh rate of 100 Hz and resolution of 1024 × 768 pixels at a distance of 60 cm from the subject. Heads were stabilized by a bite bar. Stimulus presentation and data acquisition were controlled by a Windows PC (Dell, Round Rock, TX) using the Windows 7 operating system and running the Experiment Builder display and data acquisition program (SR Research).

The EyeLink II apparatus was positioned on the head so that no part of it blocked the view of the screen. The participant's head was located 55 cm from the display monitor. Calibration of eye position was performed by having participants fixate nine locations formed in a 3 × 3 grid. At the start of each block of trials, a drift correction operation was performed that took into account recorded eye position when gaze was directed at the center of the screen.

### Experiment 1 (target with real motion)

In this experiment, subjects made saccades to an object moving in a circular trajectory centered on fixation. The target was a 1°-diameter white disk. The initial position of the target along its circular trajectory was random. Targets appeared at one of three eccentricities (4°, 8°, or 12° from central fixation) and then moved either clockwise or counterclockwise at one of five possible angular speeds of revolution: 0.125, 0.25, 0.5, 1, or 2 revolutions per second (rps). The direction of motion is easily perceived at all of these angular speeds, although the ability to track the object attentionally falters around 2 rps (Holcombe & Chen, 2012; Holcombe & Chen, 2013; Verstraten, Cavanagh, & Labianca, 2000). Therefore, there were 3 (eccentricity) × 2 (direction of revolution) × 5 (angular speed of revolution) = 30 experimental trial types. In each session, eight trials of each of the above trial types were included, for a total of 240 trials. For each of the three eccentricities, 16 control trials with a non-moving stimulus presented statically were also included. Thus, there were 240 + (16 × 3) = 288 trials in each session. Trials were intermixed, in blocks of either 36 or 18 trials. Each subject participated in three sessions.

**Figure 1.**
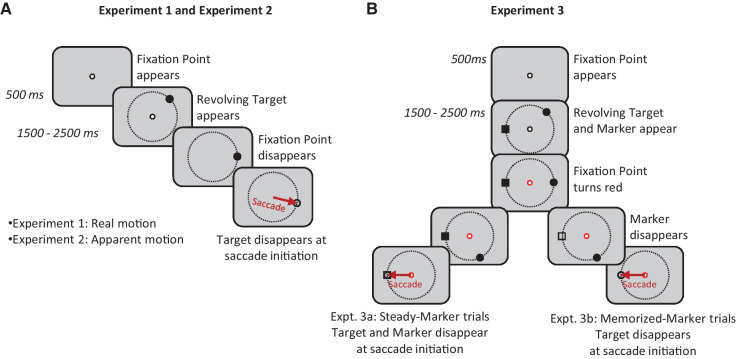
Schematic diagram of the experimental procedures. (**A**) Experiments 1 and 2. After 1500 to 2500 ms of central fixation, the fixation point disappeared, after which subjects made a saccade to a revolving target as quickly as possible. The target moved in real motion in Experiment 1 and apparent motion in Experiment 2. (**B**) Experiment 3. The procedure was similar to Experiments 1 and 2, but a stationary saccade target marker was also present on the target trajectory, and, after the fixation point turned red, a saccade was made to the location of the marker so that the eye would land on the marker at the time the target passed it. In the visible-marker trials (Experiment 3a), the marker remained visible until saccade initiation; in the memorized-target trials (Experiment 3b), the marker disappeared after 1000 ms.

For each trial type (Figure 1), trials began with the presentation of a white, square central fixation point (0.25° width). Subjects were required to fixate it within 1000 ms of its presentation. Then, 500 ms after eye fixation was detected, the peripheral stimulus appeared in motion (except in the stationary control trials, in which it appeared but did not move). The fixation stimulus remained visible for an additional 1500 to 2500 ms (time drawn randomly from a uniform distribution on each trial), during which subjects were required to continue fixating centrally. Subjects were instructed to make a saccade to the moving stimulus as quickly and accurately as possible after the disappearance of the fixation point. If subjects failed to maintain fixation on the fixation stimulus until its disappearance or if they did not make a saccade to the peripheral target within 750 ms of fixation point disappearance, all stimuli disappeared immediately and the next trial began after the intertrial period.

In order to prevent motor learning from ameliorating any dysmetria in saccade programming, we eliminated the postsaccadic motor error signal by making the saccade target disappear as soon as saccade initiation was detected. A blank, intertrial interval of 700 ms followed.

### Experiment 2 (target in real or apparent motion)

Methods were similar to Experiment 1, except that targets were presented in apparent motion (discrete steps) on most trials rather than in continuous motion (although, given the frame rate used in Experiment 1, stimuli jumped up to ∼7° in polar direction from frame to frame). Stimuli appeared at an eccentricity between 10° and 12° (chosen randomly on each trial), and moved clockwise or counterclockwise at angular speeds of 0.25, 0.5, 1.0, and 2.0 rps, either in real motion (100 Hz) or in apparent motion with jumps of 35°, 70°, or 140° in a polar direction along the circular trajectory, with target jumps occurring between adjacent monitor frames (that is, there was no interstimulus interval). As in Experiment 1, the initial position of the target along the circular trajectory was random. Also, as in Experiment 1, there were control trials with a non-moving stimulus. Therefore, there were 2 (direction of revolution) × 4 (angular speed of revolution) × 4 (real motion or jump size of apparent motion) + 1 (controls) = 33 trial types. In each session, eight trials of each of the above trial types were included, for a total of 264 trials. Trials were run intermixed in blocks of 10 to 12 trials. Each subject participated in three sessions. Other characteristics of the experiment were identical to those of Experiment 1.

### Experiment 3 (visible or memorized marker on the circular trajectory)

This experiment examined how saccades would differ (in particular, whether they would have less hypometria) if they were made to predefined locations along the target trajectory. As for the targets in real motion in Experiment 2, the eccentricity of the circular trajectory was set to between 10° and 12° (chosen randomly on each trial), and the stimulus moved at 0.125, 0.25, 0.5, 1.0, or 2.0 rps, clockwise or counterclockwise. There were three different “marker” conditions. The “no marker” trials were identical to those of Experiment 1. In the “visible marker” trials, a green square (0.5° in width) appeared at a random polar angle with an eccentricity equal to that of the moving stimulus. This static green square marker appeared at the same time as the moving stimulus first appeared. After 1500 to 2500 ms of stimulus motion, the black central fixation point changed to red. Subjects were instructed to make, after the fixation point turned red, the saccade such that it would land on the green marker at the time that the white revolving target passed over it. The “memorized marker” trials were identical to the “visible marker” trials except that the green marker would disappear 1000 ms after it appeared, and subjects were instructed to make a saccade to the memorized location of the marker at the time that the white revolving target passed over it. Control trials were included that were similar to the “visible marker” and “memorized marker” trials, except that the ordinarily moving stimulus did not move. As such, these trials resembled the well-known visually guided and memory-guided delayed saccade tasks (Fischer & Boch, 1981; Hikosaka & Wurtz, 1983). Both the target and the marker (if present) disappeared after saccade initiation.

There were 2 (direction of revolution) × 5 (angular speed of revolution) × 3 (no marker, visible marker, memorized marker) + 2 (controls) = 32 trial types. In each session, eight trials of each of the above trial types were included, for a total of 256 trials. Each subject participated in three sessions. Trials were run intermixed in blocks of 10 to 12 trials. Other characteristics of the experiment were identical to those of Experiment 1.

### Data analysis

Data analysis was performed using MATLAB (MathWorks, Natick, MA), SigmaStat (Systat Software, San Jose, CA), and SPSS Statistics (IBM, Chicago, IL). All stated statistically significant differences had *p* ≤ 0.05. One statistical approach used frequently here was to compute mean values of a particular metric (e.g., saccade gain) for each subject for each target speed and then calculate the Pearson product–moment correlation between the metric value and target speed. A statistically significant relationship between the metric and target speed would then indicate that the target speed was influencing the metric.

The dependence of saccade amplitude gain on target speed and eccentricity was assessed by computing average amplitude gain for each combination of participant, target speed, and target eccentricity and then performing a nonlinear mixed-effects estimation using the nlmefit routine in MATLAB. This constructed a regression model including both fixed and random (participant) effects. The nlmefit routine was run with its default parameters. We then compared model fits by computing *F*-ratios of model residual sum-of-square errors and comparing their Akaike information criteria (AIC, computed by the nlmefit routine), a measure of model quality incorporating both the degree of error minimization and parsimony (number of model parameters). For multiple comparisons, Tukey's honestly significant difference (HSD) test was used in the context of analyses of variance (ANOVAs) and analyses of covariance (ANCOVAs) (Hayter, 1984), and the Benjamini–Hochberg procedure was used for other multiple comparisons (Benjamini & Hochberg, 1995).

### Calculation of saccade metrics

To compute the start and end of each saccade, first a saccade velocity trace was obtained by differentiating the horizontal and vertical components of the eye position trace by a central difference algorithm implemented in MATLAB. The components were combined (via the Pythagorean theorem) to calculate the linear speed of the saccade as a function of time. To determine saccade latency, the eye position trace just after the time of the cue to make a saccade (either target appearance or fixation point disappearance; see below) was analyzed to determine the first point at which velocity exceeded 35°/s. Next, the trace was evaluated backward in time until the first point with a speed below 15°/s was found. The end of the saccade was determined in the same manner but with time reversed.

If the saccade plan updates as the target continues moving, the curvature of saccade trajectories would be modulated by the direction of target movement. We defined saccade curvature by first calculating the maximum perpendicular distance from the vector defined by the start and end points of the saccade to the actual saccade trajectory and then dividing this distance by saccade amplitude. The signed saccade curvatures were defined by the direction of saccade trajectory deviation from this vector; negative values represent trajectories deviated clockwise; positive, counterclockwise (Ludwig & Gilchrist, 2002).

## Results

We will refer to the speed of the target when reported in degrees of visual angle per second as “linear speed” to avoid confusion with “angular speed,” for which the units used are revolutions per second. The *linear speed* of a revolving target is the scaled product of the eccentricity and angular speed, whereas the *angular speed* is unaffected by eccentricity.

### Experiment 1

#### Saccadic gain

**Figure 2.**
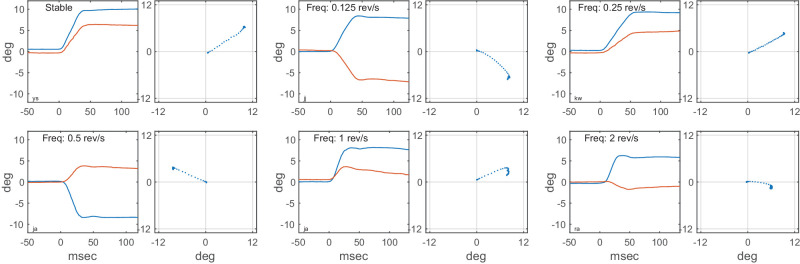
Plots of sample eye movement trajectories in time (left) and space (right), one for each of the six different speed conditions in Experiment 1. Target eccentricity in all examples was 12°. In the time plots, time is relative to saccade initiation.

**Figure 3.**
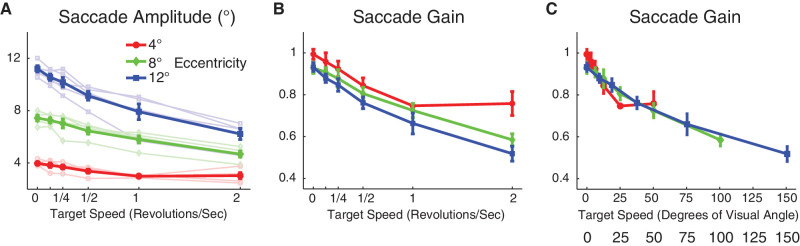
Saccade amplitude and gain as a function of target speed in real motion (Experiment 1). Thin lines indicate data from individual subjects, and thick lines indicate group averages; error bars indicate *SEM*. The target revolved around a fixation point at three different eccentricities: 4° (red), 8° (green), and 12° (blue). (**A**) Saccade amplitude is plotted as a function of speed of revolution. (**B**) Saccade gain is plotted as a function of speed in revolutions per second. (**C**) Saccade gain is plotted as a function of target speed in terms of visual angle (speed in space).

Unexpectedly, saccades elicited by the rapidly revolving stimuli were substantially hypometric. This hypometria increased with target angular speed, especially for targets at 12° eccentricity, where average gain (saccade amplitude divided by target eccentricity) dropped to around 55% for a target angular speed of 2 rps. Example saccade trajectories with a 12° eccentric target for each of the angular speeds are shown in Figure 2. The dependencies of gain on target speed and eccentricity are portrayed in Figure 3. The curves for gain versus target speed for each eccentricity partially overlap in Figure 3C, where saccade gain is plotted against target linear speed (the product of angular speed and eccentricity). This relationship between amplitude gain and linear speed appeared exponential with a non-zero asymptote. We assessed this quantitatively by using nonlinear regression (see Methods) of gain against either angular or linear speed, using models that either did or did not incorporate eccentricity (Table 1). Using linear speed as the speed measure resulted in less than half the mean squared error than did angular speed when no eccentricity term was used. This halving of squared error is a measure of the relative goodness of fit and was significantly different from 1.0 using the *F*-test, *F*(83, 83) = 0.45, *p* < 0.001, indicating that linear speed provided a better fit. Adding eccentricity to the linear speed model resulted in virtually no improvement in fit and a coefficient for the eccentricity term that was not statistically significant (*p* = 0.44), along with an increase in AIC resulting from the use of more parameters (Table 1; see Methods). In contrast, adding an eccentricity term to the angular speed model resulted in a statistically significant eccentricity coefficient (*p* < 0.001) and a clear reduction in mean squared error, although it was still nominally greater than that using linear target without eccentricity (Table 1). Note also that, as linear speed is the product of angular speed and eccentricity (multiplied by 2π), a model using only an interaction of angular speed and eccentricity is mathematically equivalent to that using linear speed by itself. In sum, although our data are far from conclusive, the most parsimonious explanation for the dependence of gain on stimulus factors is that gain is a decaying exponential function of linear target speed plus a constant.

**Table 1.** Comparison of model performance explaining the decrease of saccade gain with increasing target speed and eccentricity, where *A*_0_ = maximum saccade gain; *b* = exponential decay rate of gain with target speed; *c* = coefficient for eccentricity; *A*_1_ = asymptotic amplitude with increasing speed; *S* = speed; *E* = centered eccentricity (eccentricity – 8°, which was the mean eccentricity); Standard errors of the parameter estimates are in parentheses. Parameter estimates shown in italics are not statistically significant (*p* < 0.05). AIC, Akaike information criterion; *MSE*, mean squared error; n/a, not applicable.

		Parameter estimate (±*SE*)					
Model	Speed measure (*S*)	*A*_0_	*b*	*c*	*A*_1_	*MSE*	AIC
*A*_0_ *e* ^–^ *^bS^* + *A*_1_	Linear	0.56 (0.055)	0.0101 (0.0020)	n/a	0.39 (0.062)	0.0021	−264
*A*_0_ *e* ^–^ *^bS^* + *cE* + *A*_1_	Linear	0.55 (0.054)	0.0103 (0.0021)	*−0.0014 (0.0018)*	0.40 (0.060)	0.0020	−262
*A*_0_ *e* ^–^ *^bS^* + *A*_1_	Angular	0.40 (0.048)	0.90 (0.025)	n/a	0.55 (0.058)	0.0046	−202
*A*_0_ *e* ^–^ *^bS^* + *cE* + *A*_1_	Angular	0.45 (0.041)	0.73 (0.017)	−0.013 (0.0015)	0.50 (0.049)	0.0023	−252

We analyzed whether the hypometria depended on saccade direction. We divided our data sets into four quadrants (right, up, left, and down), with the two diagonals serving as the boundaries. The average saccadic gains across all subjects, target angular speeds, target eccentricity, and target moving direction were 0.80, 0.82, 0.81, and 0.80, respectively, for right, up, left, and down quadrants, which were not statistically different from each other (four-way ANOVA, target angular speeds × target eccentricity × target moving direction × subjects), *F*(3, 4073) = 0.62*, p* = 0.600.

For simplicity, in the analyses below we report data only for the highest eccentricity (12°) target trials, for which target motion–induced hypometria was greatest (we also analyzed data for the other eccentricities, which showed similar effects of target angular speed).

#### Amplitude variability

**Figure 4.**
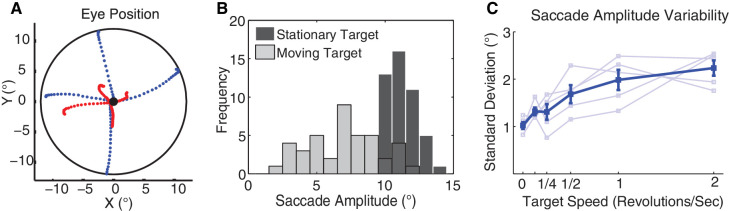
Saccade amplitude variability as a function of target velocity in real motion (Experiment 1). (**A**) Examples of actual saccade trajectories toward stationary target (blue) and high-speed moving target (2 rps, red); the black-filled circle indicates the fixation point, and the black line indicates the target eccentricity at 12°. (**B**) Histograms of saccade amplitude toward stationary target (black bars) and high-speed moving target (2 rps, gray bars) for 12° eccentricity in a typical subject. (**C**) Standard deviation of saccade amplitude as a function of target speed in revolutions per second. Thin lines indicate data from individual subjects, the thick line indicates group averages, and the error bars indicate *SEM*, all for 12° eccentric targets only.

Not only did movement of the target induce hypometric saccades but it also increased the variability of saccadic amplitude. Figure 4A shows a few examples of saccade trajectories toward a stationary target and toward a rapidly revolving target (2 rps) at 12° eccentricity. The histograms in Figure 4B show an example of one subject's distribution of amplitude of saccades toward steady targets and rapidly moving targets (2 rps) at 12° eccentricity. The saccade amplitude distribution for moving targets is noticeably broader than that for steady targets; mean standard deviation was 1.02° for steady targets but 2.2° for 2 rps, significantly larger; for two-sample *t*-test, *t*(8) = 6.8, *p* < 0.001. To quantify the effect of target speed on variability of saccade amplitude, we calculated the standard deviation of saccade amplitude for each subject for each target angular speed (Figure 4C), and averaged these standard deviations across subjects. The correlation between standard deviation of saccade amplitude and target angular speed was high (Pearson's *r* = 0.93, *p* = 0.005). These findings indicate that saccade amplitude variability increased substantially with target speed.

#### Saccade velocity

**Figure 5.**
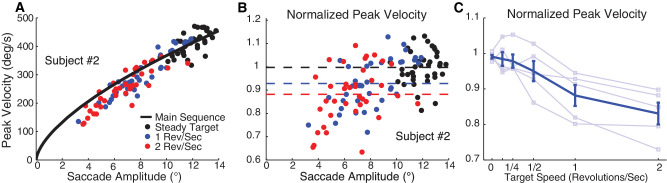
Kinematic properties of saccades to revolving targets (Experiment 1). (**A**) Saccade peak velocity (°/s) as a function of amplitude (main sequence) in a typical subject; the black line was calculated by fitting data from saccades toward stationary targets at all eccentricities. Each circle represents data from an individual trial with 12° target eccentricity (black, steady target; blue, 1 rps; red, 2 rps). (**B**) Normalized saccade velocity as a function of saccade amplitude. Dashed lines indicate the average of each target velocity. (**C**) Average of normalized peak velocity across all subjects as a function of target speed of revolution.

The consistent relationship between the peak velocity of a saccadic and its amplitude is known as the “main sequence” (Bahill, Clark, & Stark, 1975). Here, we found a systematic departure from the main sequence: The results from the 12° target eccentricity showed that saccades to targets revolving at high speeds had lower peak velocities than similar-sized saccades to stationary targets. To quantify this effect, we first estimated the main sequence of saccades toward the stationary targets in all three eccentricities for each subject separately, by linearly regressing saccade velocity on the square root of saccade amplitude (Figure 5A) (Lebedev, Van Gelder, & Tsui, 1996):

$$\mathrm{Peak}\;\mathrm{Velocity}=a\sqrt{}\mathrm{Amplitude}$$

Next, we calculated a measure of “normalized” peak velocity for saccades toward the highest eccentricity target (12°) by dividing the saccadic peak velocity by the peak velocity of a same size saccade, as defined by the main sequence curve fitted to the data for stationary targets (Figure 5B). This normalized peak velocity decreased with increasing target angular speed, with statistically significant decreases at target angular speeds of 0.5, 1, and 2 rps (two-way ANOVA target angular speed × subject), *F*(5, 1206) = 18.4, *p* < 0.001, followed by Tukey's HSD for multiple-comparison tests, which showed significant differences between the three highest angular speeds and the other speeds. We also averaged the normalized saccadic peak velocities for each target angular speed in each subject for saccades toward the highest target eccentricity (Figure 5C). The negative correlation between normalized peak velocity and target angular speed across subjects was strong and significant (Pearson's *r* = −0.9, *p* < 0.001), indicating that increasing target angular speed decreased peak saccade velocity.

The lower velocities indicate that the main sequence is different for moving targets, but in what way? The peak velocity might still have a square-root relationship with saccade amplitude, but simply with a lower proportionality constant, or it might have another relationship entirely. Visible inspection of Figures 5A and 5B suggest that the moving-target peak velocities depart from the square-root relationship, with saccades of the lowest amplitudes having the largest velocity discrepancies.

#### Saccade curvature

**Figure 6.**
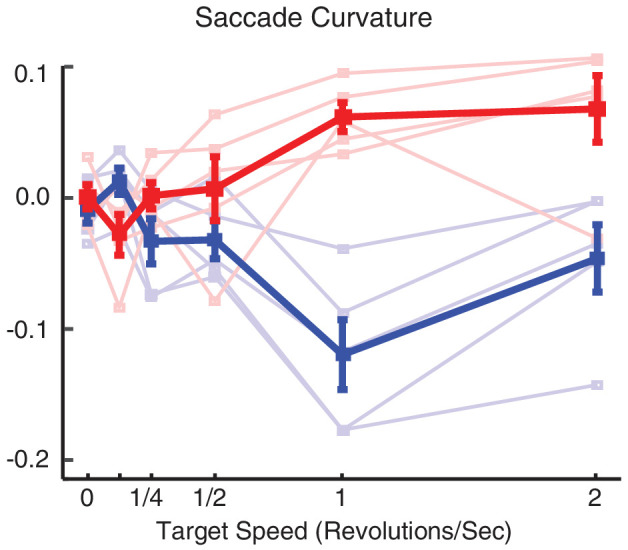
Saccadic curvature in trials with clockwise (blue) and counterclockwise (red) moving targets.

Given a moving saccade target, the saccadic system might continuously update a planned saccade motor vector during saccade programming, resulting in a program shifting in polar angle being broadcast from cortex to midbrain to eye muscles as the saccade continues, thereby producing a saccade with a curved trajectory. Another mechanism that could produce such a trajectory is the combination of a smooth pursuit component driven by the motion of the target with a fixed saccade command driven by its displacement. To test this, we assessed whether saccades curved toward the direction of motion and whether they curved more for higher target angular speeds. We then explored in more detail the effect of target speed on saccadic curvature for each direction of target motion for targets at 12° eccentricity. Figure 5 shows that saccadic curvature and target speed were positively correlated. We separately analyzed the counterclockwise and clockwise target motions (Pearson's for clockwise and counterclockwise, respectively: *r* = −0.62, *r* = 0.38; *p* < 0.001, *p* = 0.036, corrected by Benjamini–Hochberg procedure). Moreover, an ANCOVA test yielded a significant difference between the slopes: *F*(1, 8) = 7.18, *p <* 0.028 (clockwise and counterclockwise); slope = −0.04 and 0.03; intercept = 0.01 and 0.01. Therefore, saccade curvature was significantly correlated with target angular speed and its direction corresponded on average to the direction of the target motion (Figure 6).

#### Angular error

The hypometria documented above prevented interception of the rapidly revolving target with a single saccade. We also analyzed whether the direction of the saccade was appropriate. We calculated the angular (directional) bias—whether the saccades landed more in front (leading) or behind (lagging) the moving target. Ideally, a saccade would extrapolate the motion of a target when programming the saccade to estimate its future location, taking into account both the visuomotor delay (∼70–100 ms) and saccadic duration. If saccades completely compensate for these issues, then saccades should land with no angular bias. If saccades take into account neither visuomotor delay nor saccade duration, then saccades should be directed at the location the target occupied around 75 ms before the start of the saccade.

**Figure 7.**
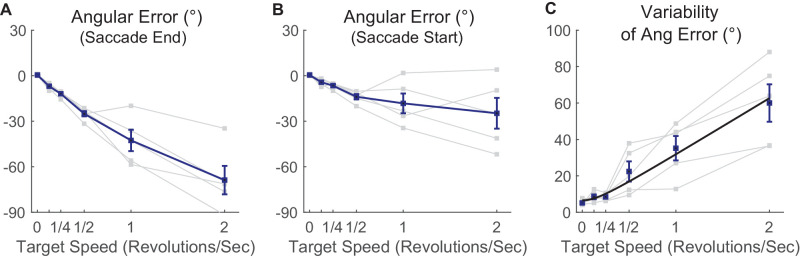
Saccadic angular error as a function of target velocity in real motion (Experiment 1). Only data from targets at 12° eccentricity are shown; thin lines indicate data from individual subjects, thick lines indicate group averages, and error bars indicate SEM. (**A**) Saccade angular error with respect to the end of the saccade. (**B**) Saccade angular error with respect to the start of the saccade. For both (**A**) and (**B**), the negative angular error corresponds to lag (**C**). The standard deviation of saccade angular error with respect to saccade start is shown. The thick line indicates curve fit (see text for details).

Saccades tended to lag the target. In other words, saccade angles corresponded to positions the target occupied before its location at the completion of the saccade. We defined angular error as the polar direction difference between the saccadic vector and target location at the end of saccade, such that, when the saccade lagged the target, this angular error was defined as negative. Saccadic angular error with respect to end of the saccade is plotted as a function of target speed in Figure 7A. The correlation between this saccadic angular error and target angular speed for the most eccentric (12°) stimuli was significant (Pearson's *r* = −0.92, *p* < 0.001) and regression estimated the slope as −34.2°/rps and intercept as −3.7°. We also ran one-sample *t*-tests to compare average angular errors with respect to the end of the saccade across subjects for each target angular speed with zero; the results indicated a significant difference for each target angular speed, meaning the saccade significantly lagged the target at every speed: *t*(4) = −7.1, −12.9, −14.3, −6.1, −7.4; *p* = 0.0026, <0.001, <0.001, 0.0038, 0.0026, corrected by Benjamini–Hochberg procedure.

These results clearly show that saccade compensation for angular target motion is incomplete at best. We next examined angular error with respect to the position of the object at the time of the start of the saccade, reasoning that finding an angular error corresponding to a delay of 70 to 100 ms would suggest that *no* compensation was being performed, as 70 to 100 ms is a rough estimate of visuomotor delay (Edelman & Keller, 1996). We analyzed this by calculating angular error with respect to the start of the saccade (Figure 7B): The relationship between angular error and target speed was roughly linear up to 0.5 rps and quite consistent across subjects, but error began to saturate and be subject to large intersubject variability at speeds of 1 and 2 rps. The linear dependence of error on delay for target speeds up to 0.5 rps implies a constant delay for low speeds, which we calculated by dividing error by target speed. For these lower speeds we inferred an average delay of 82 ms, close to that seen in the human saccadic system, as is evident in the minimum reaction time for human express saccades (Bibi & Edelman, 2009).

#### Temporal precision

Variability of saccadic angular error is presented in Figure 7C for saccades toward the highest eccentricity target (12°). This variability reflects at least two components: spatial variability, which is a constant term, and temporal variability, which is proportional to angular speed. The spatial variability reflects the basic error of the saccadic targeting system. Temporal variability is also inherent in any biological system; variation from trial to trial of afferent neural visual latency results in variation of the time that the target position is sampled from, and there will also be variation in saccade preparation, command creation, or execution time. Temporal variability could be further inflated if target position is intermittently sampled, according to some theories of the role of attention-related neural oscillations (Vanrullen & Dubois, 2011), or if the sensory temporal integration window used for saccade targeting is long. Such temporal variability results in angular error increasing with target angular speed, proportionally. In other words, temporal variance $\sigma_{t}^{2}$ will manifest in angular error as (angular speed × σ*_t_*)^2^*.* Variances add, so total variance will be the sum of the spatial and temporal variances. Thus, we modeled the standard deviation of saccade angle as

$$\begin{array}{c} \sqrt{\sigma_{x}^{2}+{\left(angular\;speed\cdot \sigma_{t}\right)}^{2}} \end{array}$$

Across the data of all participants, we fit this equation to the average of saccadic angular error variability for each angular speed for each subject using nonlinear regression (see Methods). The fit was good (*r*^2^ = 0.95; see the black curve in Figure 7C). The estimate of σ*_x_* (spatial variability) was 6.55°, and for σ*_t_* (temporal variability) it was 86.7 ms. The fits at the individual participant level were also good, with the lowest *r*^2^ across all five subjects (*r*^2^ *=* 0.95, *p* = 0.0032). However, with a large degree of intersubject variability evident, it is likely that other factors besides spatiotemporal variability contributed to the change in variability with speed, which bears further investigation.

#### Eye movement behavior before and after the primary saccade

The rapid movement of the target raises the questions of whether anticipatory pursuit occurs just prior to the saccade and whether pursuit and possibly corrective saccades occur immediately after the saccade (Kowler, Rubinstein, Santos, & Wang, 2019). Note that the revolving movement of the target makes any anticipatory movement less advantageous than if the target was moving linearly, as participants would not be able to match target velocity given that they are close to the center of the target trajectory prior to the movement. Also, as the target disappeared immediately after saccade onset for all trials, participants knew that there would be no stimulus to track after the primary saccade landed.

Nevertheless, for trials with 12° target eccentricity, we analyzed how much eye position changed in the 100-ms intervals before the saccade and after the saccade by computing the difference in mean eye position (in Cartesian space) in the first 20 ms and last 20 ms in each of these 100-ms intervals. We also determined the number of corrective saccades in the 100 ms after the primary saccade to the target.

**Figure 8.**
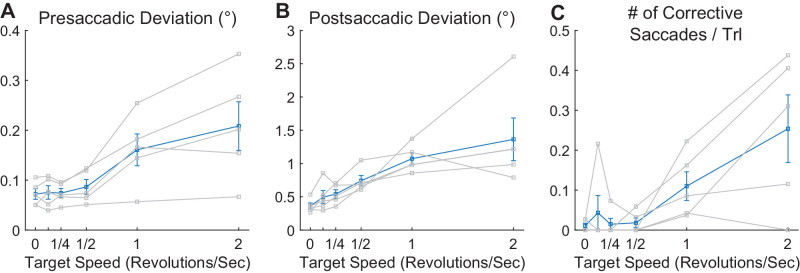
Measures of presaccadic and postsaccadic behavior as a function of target speed in real motion (Experiment 1). Only data from targets at 12° eccentricity are shown; thin lines indicate data from individual subjects, thick lines indicate group averages, and error bars indicate *SEM*. (**A**) Presaccadic deviation in the 100 ms prior to saccade initiation. (**B**) Postsaccadic deviation in the 100 ms after saccade completion. (**C**) Number of corrective saccades per trial in the 100 ms after saccade completion. See text for additional details.

We found strong correlations between target angular speed on one hand and both perisaccadic eye position change measures and the number of corrective saccades on the other hand (presaccadic eye position change: *r*^2^ = 0.48, *p* < 0.001; postsaccadic eye position change: *r*^2^ = 0.56*, p* < 0.001; number of corrective saccades: *r*^2^ = 0.48, *p* < 0.001) (Figure 8). However, these quantities were quite small, with the average presaccadic eye position excursion rising only to about 0.2° (Figure 7A), an average postsaccadic eye position excursion rising to less than 1.5° (Figure 7B), and the number of corrective saccades per trial rising to about 0.25 at the highest speed of 2 rps (Figure 7C). Note also that interindividual variability was quite high for all three measures and that, considering postsaccadic eye position shifts, the mean eye position change was much less than the approximately 11.5° of visual space the target would have traversed in that time if it still had been visible.

#### Saccade latency

Finally, it is possible that saccade amplitude gain was poor with high target angular speed because participants reacted more quickly and thus had less time to process the target motion prior to initiating the saccade (Kowler & Blaser, 1995). Note that participants viewed the revolving target for 1500 to 2500 ms before the central fixation point disappeared, which cued the participant to make the saccade. Thus, any differences in latency between speed conditions would have to be considerable to make a large relative change in viewing time. That said, it is possible that participants did not take advantage of the pre-cue period and began programming the saccade only after the cue. In any case, for the 12° target trials we assessed latency as a function of angular speed. We found that latency and speed were highly correlated (*r*^2^ = 0.45*, p* < 0.001), but that latency was *longer* for higher speed targets, with a mean latency of 210 ms for stable targets, rising to just over 300 ms for 2 rps.

In sum, results from Experiment 1 suggest that saccades toward rapidly revolving targets had dramatically smaller amplitudes and reduced accuracy than saccades to stationary targets of the same eccentricity. Saccades also tended to lag the position of the moving target. This last result contrasts with a popular interpretation of the flash-lag effect, that the visual system extrapolates moving objects to compensate for sensory latency and to successfully interact with where a moving object actually is (Nijhawan, 2008).

To sum up, compared to saccades of equivalent size to stationary targets, the saccades to rapidly moving targets had lower peak velocities, falling below the amplitude–peak velocity main sequence. Despite the hypometria and lag, saccade programming still reflected properties of the stimulus, tending to deviate in the direction of the target motion, with larger curvature for higher target speeds. The magnitude of eye movement position shifts before and after the saccade correlated positively with target speed, although these excursions tended to be small. The number of corrective saccades was also statistically significantly correlated with target speed, although the proportion of trials with corrective saccades was small. Latencies were significantly higher for higher target speeds.

### Experiment 2

Experiment 1 showed that rapidly revolving targets can induce severe saccade hypometria. In Experiment 2, we examined whether this saccade deficit resulted from a combination of the high speed and smoothness of the trajectory of the target or simply from the rapidity with which the target traveled. The real motion conditions of Experiment 1 were repeated, but trials were also run with saccade targets in apparent motion with different jump sizes, yielding rapid travel but without smoothness of motion. Because the hypometria found in Experiment 1 was greatest at large eccentricities, we focused on large eccentricities in Experiment 2, setting target eccentricity randomly to between 10° and 12° on each trial.

#### Saccadic gain

**Figure 9.**
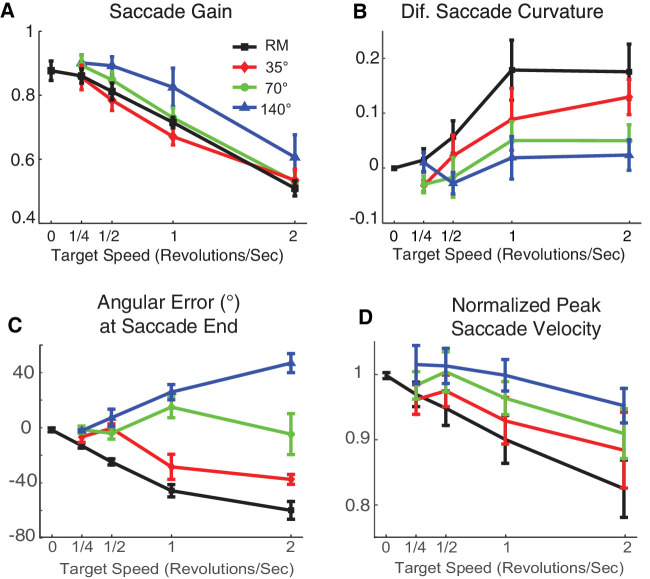
Characteristics of saccades to targets in real motion and each type of apparent motion (Experiment 2). The target moved along a circular pathway in real motion (black/thick line) and apparent motion with 35° (red), 70° (green), and 140° (blue) jumps. Error bars indicate the *SEM* across subjects. (**A**) Saccadic gain. (**B**) Difference in saccadic curvature between counterclockwise and clockwise directions. (**C**) Saccadic angular error with respect to the landing time; a positive angular error corresponds to lag. (**D**) Normalized saccade velocity.

Similar to the conditions of real motion, increasing the speed of targets in apparent motion reduced saccade gain (Figure 9A). Indeed, saccadic gain decreased with target angular speed in all motion conditions, both real and apparent (Pearson's *r* in real motion, step sizes 35°, 70°, and 140°; *r* = −0.99, −0.99, −0.98, −0.94, *p* = <0.001, 0.001, 0.002, 0.016, corrected by Benjamini–Hochberg procedure) without a significant difference between the slopes (three-way ANCOVA, dependence of saccade amplitude on target angular speed × type of motion × subject), *F*(3, 9) = 0.9, *p* = 0.464. However, the apparent motion condition with 140° jumps had significantly higher gains than the smaller jumps (three-way ANCOVA type of motion × target angular speed × subject), *F*(3, 893) = 28.9, *p <* 0.001, followed by Tukey's HSD for multiple comparison tests that showed significant differences between the 140° jumps and the others. Hypometria is thus alleviated, but only partially, with larger jump sizes, jumps so large that the target is practically jumping from one side of the screen to the other, no longer appearing to be moving in a circular trajectory.

#### Saccade velocity

Normalized peak velocity of saccades toward targets in apparent motion, like real motion, was also correlated with target angular speed (Figure 9D), although peak velocities tended to be higher for the 140° jumps, though with a correlation that just missed statistical significance. (Pearson's for real motion, jump sizes 35°, 70° and 140°; *r* = −0.99, −0.96, −0.94, −0.86, *p* = <0.001, 0.006, 0.017, 0.058, corrected by the Benjamini–Hochberg procedure).

#### Saccade curvature

Saccadic trajectories followed the moving target in real motion and small step size apparent motions. In order to simplify the statistical analysis, we subtracted the averaged saccadic curvature for clockwise from counterclockwise moving targets for each individual subject and for each target speed (Figure 9B). If the target motion cannot modulate the saccadic curvature, differences between curvatures should not be observed. The curvature difference was significantly correlated to target motion for real motion and 35° step size, but not for 70° and 140° (Pearson's for real motion, step sizes 35°, 70°, and 140°: *r* = 0.64, 0.61, 0.42, 0.19; *p* = 0.002, 0.002, 0.047, 0.349, corrected by Benjamini–Hochberg procedure; slope = 0.09, 0.07, 0.03, 0.01; intercept = 0.01, −0.01, −0.01, 0). An ANCOVA of curvature difference with target angular speed and subject as factors revealed that the saccadic curvature difference decreased as the target step size increased (two-way ANCOVA curvature difference on target angular speed × subject), *F*(3, 92) = 3.6, *p* = 0.016).

#### Angular error

The polar angular error with respect to the end of saccade was similar for real motion and 35° jumps with saccades, like those in Experiment 1, lagging the target (Figure 9C). In contrast, for saccades toward the targets in the 70° apparent motion condition there was no detected relation; for 140°, apparent motion saccades led the target in terms of polar angle (one-sample *t*-test for real motion, step sizes 35°, 70°, and 140°), *t*(19) = −7.7, −4.2, 0.2, 3.9; *p* = <0.001, <0.001, 0.7821, 0.001, corrected by Benjamini-Hochberg procedure.

In sum, rapid target movement is not the only factor causing the deficit observed in Experiment 1; smooth motion makes the deficit worse. Saccadic amplitude negatively correlated with target velocity for all types of motion, but saccades toward targets with 140° jump size were significantly larger in amplitude. Similarly, saccade curvature significantly correlated with target angular speed in real and apparent motions, but the saccades were less curved for the 70° and 140° jump sizes. Moreover, target angular speed in real motion and 35° and 70° jump sizes had an effect on saccadic peak velocity, but the correlation may be weaker or absent for the 140° jump size condition. Finally, saccades in real motion and 35° jump size generally lagged the target, but saccades in 70° and 140° jump sizes apparent motion generally led the target, although this lead was not statistically significant for the 70° jump size. These results suggest that target angular speed in smaller jump size apparent motion have similar effects on saccades as real motion. But targets with larger jump sizes (140°), a trajectory extremely different perceptually from smooth motion, elicit saccades more like those of stationary targets.

### Experiment 3

In Experiments 1 and 2, subjects likely attentionally tracked, or attempted to track, the rapidly revolving stimulus before targeting it with a saccade. Experiment 3 addressed whether simply attentionally tracking a revolving stimulus could result in saccade hypometria, or instead whether explicitly *targeting* a revolving stimulus was necessary. To distinguish between these possibilities, we modified the task of Experiment 1 to require subjects to intercept the disk with a saccade toward a predefined, fixed location on its trajectory. The location was specified at the beginning of the trial either by a briefly appearing marker (memorized marker) or by a continuously visible marker (visible marker). To provide comparison data, in this experiment we also included conditions without any marker (non-marker, identical to the real motion conditions of Experiments 1 and 2).

#### Saccadic gain and velocity

**Figure 10.**
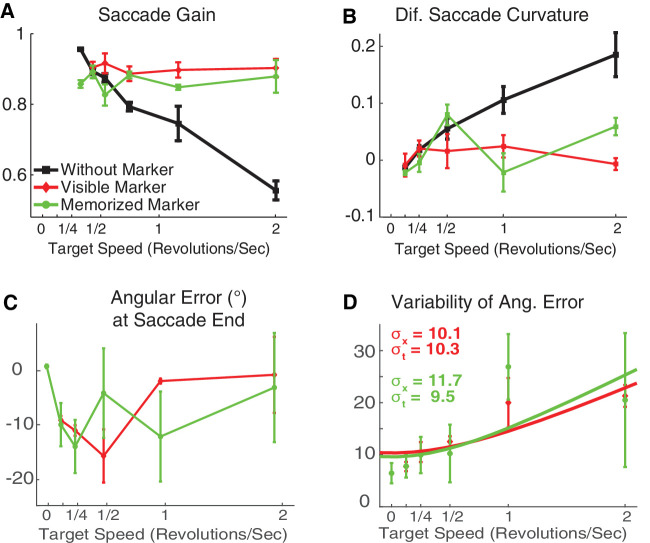
Saccade properties as a function of target velocity toward moving targets (thick/black), stable marker (red), and memorized marker (green) (Experiment 3). Error bars indicate *SEM* across subjects. (**A**) Saccadic gain. (**B**) Saccadic curvature. (**C**) Saccadic angular error. Negative values indicate that saccades landed to the side where the moving target approached the marker. (**D**) Variability of angular error.

Both the visible marker and the memorized marker alleviated saccade hypometria considerably, yielding saccades with characteristics similar to those for a stationary target. Unlike in Experiments 1 and 2, we did not find any significant correlation between disk angular speed and saccadic properties such as saccadic gain (Pearson's in visible and memorized marker, respectively: *r* = −0.14, 0.175; *p* = 0.811, 0.739) and peak velocity (*r* = 0.49, 0.11; *p* = 0.400, 0.850). But, when no marker was present, as in Experiments 1 and 2, saccades were more strongly hypometric and more curved and had slower peak velocity for high-velocity disks (Pearson's *r* = −0.97, 0.99, −0.84; *p* = 0.001, <0.001, 0.034, respectively, corrected by Benjamini-Hochberg procedure) (Figure 10A). Thus, it appears that the presence of the rapidly revolving disk is not sufficient to cause saccade hypometria, but that the inability to prepare a saccade with a specific vector is also necessary.

#### Curvature

Target motion appeared not to induce curvature of saccades when saccades were aimed at the visible or memorized marker. Results from the previous experiments showed that saccades toward the moving targets, with real or apparent motion, deviated in the direction of the target motion (Figure 10B). Similarly, in this experiment, for the *non-marker* trials the difference between curvatures of saccades made toward clockwise and counterclockwise moving directions was correlated to target speed. In contrast, we did not find any significant correlation between the saccadic curvature and target speed in trials where the saccades were toward the marker (Pearson's in non-marker, visible, and memorized marker, respectively: *r* = 0.84, −0.08, 0.31; *p* = < 0.001, 0.724, 0.254, slope = 0.10, 0, 0.02; intercept = 0, 0.01, 0). In accordance with this tendency, an ANCOVA test showed that the slope of saccadic curvature difference as a function of target speed was significantly different from zero only for *non-marker* trials (three-way ANCOVA curvature difference on target angular speed × marker × subject), *F*(2, 53) = 11.2, *p* < 0.001, followed by Tukey's HSD for multiple-comparison tests that showed significant differences between the slope for trials without marker and trials with visible or memorized markers.

#### Angular error

Saccades landed close to the marker both when it was visible and when it was memorized. We defined “marker angular error” as the difference in polar angle between the location of the marker and the saccade endpoint. To determine whether the direction of the moving stimulus influenced this error, we used signed values, with positive values indicating that the saccade landed to the side of the marker in the direction of motion, and negative values indicating that the saccade landed opposite the direction of motion. Across all subjects and speeds of the moving stimulus, the average marker angular error was very small, at −0.56° and −1.64° in visible and memorized marker trials, respectively. Neither value was significantly different from zero: one-sample *t*-test, *t*(24) = −0.7, −2.3; *p* = 0.439, 0.056, corrected by Benjamini–Hochberg procedure.

The more critical question here was the timing of the saccade. Could saccades be timed such that the saccade landed at or near the moving stimulus as it passed by the marker? We defined “moving stimulus angular error” as the difference in polar angle between the saccade endpoint and the position of the moving stimulus at the time the saccade landed. Errors were positive if saccades landed ahead of the moving target and negative if they landed behind it. Moving stimulus angular errors were small and, although significantly different from zero, one-sample *t*-test: *t*(24) = 2.5, 4.5; *p* = 0.018, <0.001, corrected by Benjamini–Hochberg procedure, varied little with disk speed, suggesting that errors simply resulted from static errors present during ordinary saccade tasks but are biased so as to lag behind the moving stimulus (Figure 10C). Participants’ success at having their eyes land near the marker very near the time that the moving target passed by it implies that participants timed their saccade quite well—taking into account both saccade programming time and duration to anticipate when the object would reach the marker.

The variability of the angular error as a function of speed in the marker conditions is shown in Figure 10D. The curve fit designed to partial out the spatial and temporal precision suggests that most of the variability is due to spatial imprecision, with the temporal imprecision being remarkably good—about 10 ms in both conditions. This is in contrast to the task of pressing a button at the moment a revolving object reaches a landmark, for which Linares et al. (2009) found over 50 ms of imprecision. However, the curve fit to the current data is noticeably poor, with the data also being consistent with a dependence on target speed that saturates at high speeds, so we believe that our study did not succeed in estimating temporal imprecision.

The pattern of performance here presents an important contrast with the experiments without a marked location to make a saccade. Without a marker, saccades landed behind the target; whereas, with a marker, saccades landed near the marker slightly before the stimulus reached the marker. Evidently, participants extrapolate object motion robustly when given a static reference location (the marker), but do so much less when attempting to saccade to a moving object without a marked location on its trajectory.

## Discussion

### Characteristics of saccades to rapidly revolving targets

These experiments show that rapid circular target motion can result in saccades that are severely hypometric. This finding is particularly surprising given the delayed nature of the task. Subjects had well over a second to plan a saccade and should have been able to generate a saccade that, at the very least, landed close to the trajectory of the target. The hypometria that resulted from making saccades to rapidly revolving targets did not seem to result from programming a smaller amplitude saccade. If it had, then one would expect the peak velocity of the saccade to be appropriate for the amplitude of the saccade made. Instead, the peak velocity of the saccades was lower that than that of similar-sized saccades to non-moving targets.

It is unlikely that the unusual saccade behavior we observed resulted from a superposition of position displacement and motion signals feeding into the saccadic system. One might posit that, when faced with a rapidly revolving target, a reasonably normal saccade program is prepared, one that would achieve the correct amplitude, if not the correct direction. Potentially, stimulus motion might also provide a smooth pursuit-like signal (Leigh & Zee, 2015) that would be added onto the “standard” saccade command. Consistent with the addition of such a pseudo-pursuit signal, we did find that saccades lagged the target but curved toward it. However, we also found extreme hypometria and abnormally low peak velocity, phenomena that are difficult to explain by the addition of a motion signal to a normal saccade command. Moreover, the saccades landed at an angle well behind the moving target, suggesting only a small (if any) contribution of a motion signal. The increase in saccade curvature observed in this study may also result in part from the planning of a sequence of saccades toward the moving target, as recent work has demonstrated that the first saccade in a sequence of saccades is typically more curved and skewed toward the direction of the next saccade (Azadi, Zhu, & McPeek, 2021).

In these experiments, we made the visual target disappear upon saccade initiation. Although the hypometria may have been partially alleviated by visual feedback across trials, the lack of feedback here cannot explain our results because saccade hypometria was greatly diminished in our control condition in which subjects make saccades to a non-moving target, which also disappeared upon saccade initiation.

### Comparison to previous work on tasks with moving targets

The original motivation for this work was to compare the ability of the saccadic system to track moving targets with that of covert attentional tracking and localization tasks. However, in Experiment 1 we discovered that saccades were severely hypometric at target speeds far below the 2-rps limit for covert attentional tracking. This complicates comparison of our results to those from tasks requiring explicit judgments of the location of a target at the time of a cue (Linares et al., 2009). Our results showed that saccade endpoints lagged the target in polar angle even for low speeds, with an error proportional to target speed for low speeds, indicating a constant delay similar to the visuomotor delay for saccade generation, whereas at high speeds saccade lag was highly variable across subjects.

These findings are not what some would have predicted from a theory popular in flash-lag effect literature, that the visual system compensates for sensory latencies in its estimation of the position of moving objects to facilitate successful interaction with moving objects (Nijhawan, 2008). With our stimuli, saccades consistently lagged moving objects, so, if the brain does seek to overcome sensory and motor latencies, it is not successful. Thus, such compensation is not hard-wired to occur correctly for the present task. With training, action routines specific to certain circumstances and/or stimuli could nevertheless eliminate systematic errors and improve precision (e.g., Brenner & Smeets, 2015).This may explain the competence of many people in ball and racket sports.

Previous studies of saccades to moving targets have found evidence that target motion is taken into account during saccade programming, although monkeys, which generally receive more training in these tasks than humans, appear to be better at this (Fleuriet et al., 2011; Guan et al., 2005; Keller & Johnsen, 1990; Keller et al., 1996) than humans, whose ability to use target motion appears to depend upon viewing the moving target prior to the cue to make the saccade. (Etchells et al., 2010; Gellman & Carl, 1991; Ron et al., 1989). Virtually all of these studies used isodirectional stimuli (where target motion was in or opposite the direction of the target jump). One exception is a monkey study (Fleuriet et al., 2011), in which the target jumped from the center to the periphery of the screen and then moved in a straight line orthogonal to the jump. They found normal saccade kinematics and slight hypometria that increased with target speed up to their highest of 21°/s. The slight hypometria we found for low speeds is similar, although our stimuli moved circularly, and saccades were made only after an instructed delay. Other than the study of Keller et al. (1996), which demonstrated that, with very high linear target speeds of 40 to 60°/s, saccades could have lower velocities, occasionally with two velocity peaks in time, previous studies seem to have little or no bearing on our finding of massive hypometria at high target speeds, as they had maximum stimulus speeds much lower than ours.

The results of Experiment 1 indicated a delay of around 90 ms with respect to the start of the saccade, which is very close to the minimal saccadic visuomotor delay observed for human express saccades (Bibi & Edelman, 2009). This finding suggests that the saccadic system programmed a movement toward the current representation of target location, with only partial accounting for the target angular speed and saccade duration.

### Possible neural mechanisms underlying saccade hypometria

The saccade system clearly performs poorly when targeting rapidly revolving stimuli. What is the neural basis of this failure? It does not appear to be due to failures in brain mechanisms regulating attention, as the deficits occur at speeds well below limits on attentional tracking of the moving target or judging its instantaneous position. Moreover, the limit on attentional tracking is set by revolutions per second (Holcombe & Chen, 2012; Holcombe & Chen, 2013; Verstraten et al., 2000), whereas the hypometria documented here appeared to be determined more by linear speed. With the poor saccade performance occurring for moving object speeds that are not associated with a corresponding deficit in explicit localization (Linares et al., 2009), it may instead be specific to the interface between vision and saccade planning or execution.

The superior colliculus (SC) plays an important role in the spatial coding and triggering of saccades (Leigh & Zee, 2015). It is thus worth considering whether the spatiotemporal dynamics of SC activity might mediate the severe hypometria found here. From single unit recordings it is known that the SC codes for saccades via a population of neural activity and that the SC is laid out as a spatial map, with different locations corresponding to different saccade vectors. Thus, when a saccade is about to be executed, a substantial portion of the SC becomes active, with the location of peak activity representing the saccade vector. In terms of saccade polar angle, the spatial profile of SC activity for a given saccade can be approximated by a Gaussian. However, it is also known that intermediate layer neurons in the SC, which trigger saccades via their projection to the saccadic burst generator in the brainstem, also have visual responses (Leigh & Zee, 2015).

If we assume that (1) activity in the human SC is similar to that observed in the monkey SC and (2) the visual response in the SC to a rapidly moving target can be predicted linearly from the response to a static target, then we argue that the spread of activity on the SC caused by such motion cannot explain the saccade hypometria. Given that the target is sweeping continuously across space prior to the saccade, it is likely that a transient, high-frequency component of the visual response in intermediate-layer cells is present at the time of the saccade. Indeed, the visual response in the SC can directly trigger a saccade, as it does for express saccades (Edelman & Keller, 1996), which have ultra-short reaction times (80–110 ms). Thus, this visual response is likely to play the predominant role in triggering the saccade to the revolving target. Edelman and Keller (1998) showed that, when two targets appear at the same eccentricity but separated in polar angle by 45°, saccadic responses tend to land somewhere between the two visual stimuli, with amplitudes only slightly smaller than the eccentricity of the targets, resembling a vector average of saccades made to each of the two stimuli. Moreover, such *averaging saccades* can also be express saccades, directly triggered by the visual stimuli, and such express saccades are produced by a single broad mound of visual-evoked activity in the SC, whose shape reflects the spatial summation of the mounds corresponding to the two targets, although the peak of the combined mound is somewhat less than what the spatial summation would predict (Edelman & Keller, 1998).

Could such spatial averaging predict the hypometric saccades we observe? In the monkey SC, the duration of the high-frequency visual response is ∼25 ms (e.g., Edelman & Keller, 1996). With the fastest target speed in the present experiment, the target travels 2 rps × 0.025 s = 0.05 revolutions, or 18° of polar angle in 25 ms. Note that, because 18° < 45°, the spatial extent of the SC activity resulting from the revolving stimulus should be less than that caused by two targets separated by 45°. Thus, the saccades produced by the revolving stimulus should be *less* hypometric than averaging saccades. That they are not suggests that SC activity cannot explain the hypometria we observe. Of course, it is possible that the rapidly moving visual target elicited activity in the SC that is somehow unlike that elicited by multiple static visual stimuli. We thus cannot rule out that abnormal SC activity may have produced the saccade hypometria we observed.

However, note also that vector averaging does not predict our finding that saccade gain appeared to depend more on the linear speed of the target (in degrees of visual angle per second) than on change of target direction (revolutions per second) (Figure 2C). With the simple averaging hypothesis, the relation between gain and the *angular* speed (in revolutions per second) should be independent of target eccentricity; instead, we found that gain decreased with higher target eccentricities, whereas the relation between gain and target *linear* speed was largely independent of target eccentricity. Moreover, saccades not only were short in amplitude but also tended to have reduced velocity compared to similarly sized saccades to stationary targets. This finding is not consistent with the idea that a normometric (at least in terms of saccade amplitude) saccade was programmed following an averaging process.

Another possibility is that the downstream brainstem and cerebellum may be the source of the hypometria. Indeed, the SC activity preceding saccades to a moving target does not seem to take into account target motion, leading to the idea that a signal corresponding to target motion bypasses the SC and affects neurons in the brainstem saccade generator and the cerebellum (Keller et al., 1996). There is evidence that the fastigial oculomotor region (FOR) of the cerebellum plays a role in saccade kinematics, particularly in ensuring that the saccade lands on target (Fuchs, Robinson, & Straube, 1993; Goffart, Chen, & Sparks, 2004; Joshi & Das, 2013). Motion of a tracked target that is very rapid and persists may overwhelm this cerebellar circuit, producing the saccade hypometria we observed. This may also explain the unusual saccade kinematics and the dependence of the hypometria effect on linear speed, not angular speed. Physiological recordings in the brainstem or FOR in monkeys performing this task could shed light on this hypothesis. Note that, per Experiment 3, the rapid shifting of the stimulus does not in and of itself inhibit the saccade generator, nor does tracking it perceptually. Instead, the moving stimulus must be targeted by the saccadic system for the hypometria to occur.

### Implications for volitional control of saccadic eye movements

According to the limits on visuospatial processing as they previously were understood, in our task the saccadic system should have been able to isolate an individual location for targeting, even at our fastest stimulus speeds, particularly because the delayed task enabled subjects to view the targets for over a second before movement execution. Even if the programming of saccade angle could not be done precisely, the voluntary saccade system ought to be able to program a saccade with correct magnitude. Yet, even the experienced participants in these experiments exhibited severe saccade hypometria, even when individual locations were salient, as with the apparent motion in Experiment 2. Only when a specific alternative location was provided (Experiment 3), so that the moving object, while setting the timing of the saccade, was not itself targeted, could subjects make roughly normometric saccades.

The participants’ inability to make normometric saccades to a moving object suggests that, when a moving object is targeted, even voluntary saccades are not under full voluntary control and can be inextricably corrupted by the presence of visual stimuli, to the extent that the resulting saccades lack any sense of goal directedness, at least in any conscious sense. Our data suggest that either the saccadic system may not always be able to program a saccade to a visual stimulus, or the saccade system is in particular circumstances incapable of executing such a program. This impaired performance in a task in which the saccade is initiated voluntarily suggests that, in a visual world, the classification of saccades as either reflexive or voluntary may be simplistic, as the continuing flow of afferent stimulation may override volitional commands despite our best efforts.

## Conclusions

These experiments show that, in a delayed saccade task, saccades made to a target undergoing rapid circular target motion, whether real or apparent, can be severely hypometric and low in peak velocity. These deficiencies are largely ameliorated when participants can intercept the stimulus with their eyes by targeting a particular location along the trajectory. These results indicate that motor output can be inextricably bound to sensory input to its detriment, even during a highly voluntary motor act.
